# Emerging themes in food security: environmental justice, extended families and the multiple roles of grandmothers

**DOI:** 10.1186/s12939-018-0856-3

**Published:** 2018-09-12

**Authors:** Ethel Alderete, Lauren Sonderegger, Eliseo J. Pérez-Stable

**Affiliations:** 1 Universidad Nacional de Jujuy, Consejo Nacional de Investigaciones Científicas y Técnicas, Instituto de Ciencia y Tecnología Regional. Currently at Centro de Investigaciones Sociales y Regionales (CISOR), Independencia 575, 4600 San Salvador de Jujuy, Argentina; 2 San Francisco School of Medicine, University of California, San Francisco, USA; 3 Present address: Swedish Family Medicine Residency Cherry Hill, 550 16th Ave, #400, Seattle, Washington 98122 USA; 4 Division of Intramural Research, National Heart, Lung and Blood Institute, and Office of the Director, National Institute on Minority health and Health Disparities, National Institutes of Health, 6707 Democracy Boulevard, Suite 800, Bethesda, MD 20892-5465 USA

**Keywords:** Food insecurity, Water insecurity, Environmental contamination, Caregiving grandmothers, Childhood development, Primary health care

## Abstract

**Background:**

Pre- and perinatal nutritional status defines the development of adult metabolism and energy balance in humans. Young children in poor households are disproportionately more vulnerable to food insecurity given the cumulative impact of chronic stress on susceptibility to chronic diseases as an adult. Qualitative studies focusing on the experience of food insecurity in Latin America are scarce. In Argentina, although socioeconomic indicators improved in the aftermath of the 2001ecomomic crisis, the disadvantaged provinces in the north continue to bear the burden of historical inequities. The study was conducted among Primary Health Care patients in the city of San Salvador de Jujuy, Argentina. It analyzes environmental and household level stressors through the narratives of mothers with young children living with food insecurity, from the perspectives of eco-developmental conceptual frameworks.

**Methods:**

We conducted 11 semi-structured interviews with mothers of children < 1 to 6 years of age who participated in maternal-child health programs in Primary Health Care clinics and lived in food insecure households. Interviews focused on the environmental context and the resources and processes for obtaining and preparing daily meals.

**Results:**

The Eco-bio-developmental (EBD) framework and the vulnerability-assets approach, provided a basis for conceptualizing the significance of findings. Our results indicated the need to understand pathways in the association of stressors, vulnerability and ill health, as well as the mitigating role of social relationships. For example, understanding the link between the stress of being exposed to environmental contaminants and the capacity to overcome food insecurity, or developing strategies to integrate the support provided by kinship networks like extended families into food security programs. The results also indicate the importance of developing support mechanisms for vulnerable family members like grandmothers in food insecure households who play instrumental roles as providers and caretakers of younger relatives.

**Conclusion:**

The empirical evidence generated by this study may inform community based strategies and public health policies to address food insecurity in vulnerable population groups who face health effects from multiple stressors.

## Background

Food security constitutes an essential aspect of human health. It refers to having an adequate diet resulting from balanced food access and nutritional requirements. Calvo and Aguirre [1] defined food security as the right of all peoples to a culturally and nutritionally adequate and sufficient dietary intake. Food security reflects three fundamental elements of availability, access and consumption. While income is a principal associated factor, household level behaviors and practices regarding the selection, the acquisition and the distribution of food, are also significant contributors to dietary patterns [2].

The effect of poor food security impacts not only on insufficient nutritional intake but also on over-nutrition leading to obesity and other nutritional changes that contribute to the development of chronic diseases. Young children in poor households are disproportionately more vulnerable to food insecurity especially given the impact of chronic stress on susceptibility to chronic diseases as an adult [3]. Hales and Barker [4] proposed that pre- and perinatal nutritional status programs the human organism’s adult metabolism and energy balance in early development. The consequences of inadequate food intake become apparent through indirect and delayed manifestations that may be irreversible. These consequences refer to alterations in physical and mental development, abnormal changes in body weight with deficiencies and excess, acute and chronic morbidity, limitations in academic performance and productivity, and mortality in all age groups [5–8].

Despite the increase in food production globally, nearly 800 million people in the world lack sufficient food to conduct a healthy and active life [9]. Food insecurity is largely due to inequalities in the distribution of resources [10]. Key characteristics of households most likely to experience food insecurity are, those with low-income, single-woman headed households, minority race, lower education levels, and a greater number of children and/or other household members [11].

Qualitative and quantitative research conducted in different locations around the globe produced evidence on coping strategies used by households facing food insecurity. Commonly mentioned strategies include eating low cost, dense in calories food of poor nutritional value; eating little variety of food items, smaller servings or fewer meals during a day; having some household members eating less to favor others; and borrowing money to purchase food [12–14]. Studies also unveiled subjective experiences of food insecurity, including negative feelings like anxiety, preoccupation, sadness or guilt [15–21].

### Food insecurity in Latin America

Food insecurity in most of Latin America is more common than in the United States where rates ranged from 12.7% countrywide to 32.8% among households living under the poverty line in 2015 [22]. Food insecurity was present in 81% of 794 low income households with children in Quito, Ecuador [23]; in 76% of 7187 households in the state of Nayarit, Mexico [24]; in 75% of 443 households in a Nicaraguan population sample [25]; in 67% of 6591 households with members over 59 years of age in Mexico [26]; in 49% of 3920 urban households and 45% of rural households in a Brazilian nationally representative sample [27]; and in 39% of 128 households of Los Morochucos district of Perú [28].

The national survey of urban households conducted in 2002 in Argentina indicated that 17.5% of the homes had experienced hunger [29], while a study conducted in nine provinces of the disadvantaged northern region in 2004, showed that 69.5% of the homes had a member who had experienced hunger, and the perception of hunger was associated with stunting in children [30]. More recently food insecurity was reported by 31.5% of 592 households in the city of Santa Fe [31] and by 11.2% of 5712 households from other main urban areas [32]. Few qualitative studies were conducted in Latin America or with a population of Latin American origin. Bernal et al., [15] examined the experience of children 10 to 17 years of age in schools of the Mirada state in Venezuela, reporting that they had cognitive and emotional awareness of the effects of food insecurity in their homes. Lindsay et al., [21] showed that socioeconomic factors and food insecurity influenced mothers of pre-school children’s feeding practices, in the city of Buenos Aires, Argentina. Among women of Mexican origin living in the United States, Dean et al., [20] inquired about the repertoires for coping with material hardship reporting reliance on inexpensive staple foods.

### Theoretical framework

The American Academy of Pediatrics (AAP) proposes an eco-bio-developmental (EBD) framework for understanding the evolution of an individual’s strengths and risks for health over the lifespan [33]. This framework has been used to understand the complex causes of food insecurity [34] arguing that food insecurity is about much more than hunger or caloric requirements, with a variety of issues impacting on the capacity of families to provide adequate nutrition to all its members. An emerging body of evidence shows that being exposed to different forms of adversity designated as toxic stressors, can impact on behaviors and choices, and shape the ability to perform adult roles in food insecure families, particularly when the exposure to these stressors is transmitted across generations [35].

On the other hand, the vulnerability and assets framework [36] focuses on identifying what the poor have, rather than on what they are lacking. Studies have shown that the poor manage a variety of resources and that asset management affects household poverty and vulnerability. Assets are categorized as tangible resources such as labor and human capital or housing, and intangible resources, such as household relations and social capital. Furthermore, the capacity to respond to stressors in the external environment depends on intrahousehold relationships and on community level trust and collaboration [37]. Analyzing vulnerability involves identifying not only the threat but also the responsiveness in exploiting opportunities and in resisting or recovering from negative effects. Resilience is reflected in the assets that individuals, households, or communities can mobilize and manage in the face of hardship. Thus, interventions should focus on promoting opportunities to ensure an effective use of assets, as well as removing obstacles [38, 39].

Based on the premises of the EBD framework and the vulnerability/assets approach, this study analyzed the narratives of mothers of small children living in households with food insecurity in Northwest Argentina. We examined negative aspects and positive contributions, limitations and resources of 1) the environmental context, 2) the household level socioeconomic characteristics and 3) the individual level roles.

## Methods

### Study site

Marked regional differences in socioeconomic and health indicators exist within Argentina. With the economic crisis triggered in 2001 the percentage of people living under the poverty line reached 54.3% countrywide, with differential distributions between affluent and poor regions of the country. Although socioeconomic indicators improved in the aftermath of the crisis, the disadvantaged provinces in the north continue to bear the burden of historical inequities [40]. This study was conducted in 2015 in San Salvador de Jujuy, the capital city of the Province of Jujuy, Argentina, a socioeconomically disadvantaged setting of the Northwest region. The nutritional profile of children in this area has been historically characterized by high prevalence of low weight and stunting. However, child overweight and obesity rates increased significantly in recent years [41, 42].

### Sampling and procedures

The study was conducted among mothers of children < 1 to 6 years of age who were clients of maternal-child health programs in Primary Health Care Clinics (PHCC) in San Salvador de Jujuy. We included families that were facing severe daily life economic hardships. Rather than seeking representativeness of the population attending PHCC, we strived to reach the comprehension of singularities. The premise is that gaining knowledge on singular cases, will contribute to the implementation of public health actions focused on the least visible and most vulnerable social groups, with the goal of extending social inclusion and reducing health inequities. Therefore, we implemented the following sampling strategy using information from previous research [42]. Among 6 randomly selected PHCC, we identified 3 with the highest percentages of families with food insecurity (23.5%, 21.6%, 20.6% compared to 12.7%, 14.7%, 6.9%). We then used a theoretical sampling strategy to recruit mothers living in food insecure households [43]. Eleven mothers who responded positively to food insecurity screening questions were invited to participate [44]. Participants received an informed consent sheet and the research was approved by the Ethics Committee of the Ministry of Health of the Province of Jujuy. One of the authors conducted the interviews in the homes, with mothers of different demographic characteristics.

### Interview themes

Themes were developed based on a review of the published literature and local considerations about the study site. Questions were asked about the neighborhood, access to public services, the constitution of the household, household economic resources, food purchasing and preparation practices, roles of family members and strategies to deal with food scarcity. The standardized observations of the environment, of the housing characteristics and the context of the interview were recorded in field notes.

### Demographics

Participants were asked their age, occupation (employed in a formal or informal job, student or housewife), number of children born alive, and about the number and category of all household members. Households constituted only by parents and their children were defined as nuclear families versus households with additional persons. The presence of partners included husbands and informal relationships. Ascertaining the presence of grandmothers in the home included the maternal and paternal lineages. The number of household members included adults and children.

### Data analysis

The frequency of demographic characteristics was calculated using the STATA software. The interviews were digitally recorded and transcribed. During the analysis, we followed an inductive reasoning, using the narratives to build interpretations and meanings. We used a case-based approach to examine individual roles, household characteristics and singularities, and the contextual scene [45]. The coding scheme was developed manually and with the assistance of ATLAS.ti (6.2). We conducted an open scheme codification to identify organizing concepts and categories. We then analyzed each category in detail, cross checking the coding and the interpretation of data between two independent analysts. Content disagreements were discussed and the emerging insights provided for refining coding frames [46]. Emerging themes were diverse and encompassed environmental contamination, lack of urban infrastructure and public services, the roles of household members, positive and negative coping strategies, and food purchasing and consumption patterns.

## Results

Table 1 summarizes the demographic characteristics of participants, 45.5% were 18 to 25 years of age, 54.5% had three or more children, the majority (54.5%) did not work or study, and 36% lived in a nuclear family household. A male partner was present in the household of 63.3% of participants, grandmothers lived in 36.6% of the households and 45.5% of the households had more than five members.

**Table 1:** Sociodemographic characteristics of mothers with children < 1 to 6 years of age (*N* = 11) interviewed in S.S. de Jujuy, Argentina, 2015

Sociodemographic characteristics of mothers	N (%)
Age in years	
18–25	5 (45.5)
26–47	6 (54.5)
Occupation	
Housewife	6 (54.5)
Student	3 (27.3)
Employed	2 (18.2)
Number of children	
1–2	5 (45.5)
3–6	6 (54.5)
Nuclear family household	4 (36.4)
Male partner living in the home	7 (63.6)
Grandmother living in the home	7 (63.6)
Households with > 5 members	5 (45.5)

In the following paragraphs we present the themes emerging from women’s narratives, encompassing contextual, family and individual level issues. Table 2 shows a summary of the themes organized into socio-environmental stressors, negative coping strategies and buffering-protective resources.

**Table 2:** Themes from Semi-Structured Interviews with 11 mothers of children in food insecure household, Jujuy, Argentina, 2015

**Socio-environmental Stressors**	
*Limited financial resources*	
“Last year my husband lost his job, we started then to reduce [food expenditures] because we have to pay the electricity, water…” (40 yrs., housewife)	
“The last days of the month, we do not have enough money to buy food, until the next month when we get paid”. (25 yrs., 3 children, housewife).	
*Environmental contamination*	
“There is also the smell from the paper mill and other factories, it depends on the direction of the wind, we wake up in the morning and the house is full of smoke”. (47 yrs., 6 children, housewife)	
*Lack of urban infrastructure*	
Interviewer: is this water safe to drink?	
Respondent: sometimes [it makes us sick] because it has insects, many things, it is dirty…but we strain it with a strainer or we tie a cloth to the pipe (21 yrs., housewife)	
*Limited food access*	
“There are no food stores here, there is one in [a nearby neighborhood], it is far. (21 yrs., 3 children, housewife)”.	
**Negative Coping Strategies**	
*Rationing of food*	
“We buy cheaper things. Sometimes the vegetables are very expensive then we cannot use vegetables…we replace them with other things...when we do not have meat we eat hotdogs, or we make pasta with sauce”. (25 yrs., 2 children, housewife).	
*Selective intake reduction*	
“I eat less, so the children and also my husband can eat more, three or four days per week”. (21 years, 3 children, housewife)	
**Buffering-Protective Resources**	
*Local markets of the informal economy*	
Respondent: We buy in the market [open air market] they bring [the vegetables] directly from the farms, they bring fresh vegetables	
Interviewer: What do you buy in the market?	
Respondent: Carrots, tomatoes, lettuce, potatoes, peas, all types of vegetables, also some fruit, apples, oranges, bananas, peaches and also pasta, corn meal, rice, oil, salt whatever is needed for cooking.	
*Pooling resources among neighbors*	
“Sometimes families come from Bolivia with lots of children, what we do is, on Sundays we go [to the market] and buy bags of potatoes, pasta, eggs. Then we bake bread”. (26 yrs., 2 children, employed)	
*Extended family support*	
“My brother in law has a job, so sometimes we have an extra income, on a weekend so we have money for food…he was unemployed and not long ago he found a job”. (33 yrs., 2 children, housewife)	
*Financial and nurturing role of grandmothers*	
Interviewer: who pays for the food?	
Respondents: my mother, she is retired and receives pension for being the mother of 7 children (39 yrs., 5 children, employed)	
“My mother decides what we are going to eat because she knows best, she knows what we are going to buy”. (25 yrs., 2 children, student)	

### Socio-environmental stressors

#### Household income

The families in this study confront precarious economic conditions resulting from a reduced capacity to generate income. Unemployment or informal and irregular work characterize their occupational condition.

*My husband does not have a good job…[the income] is not enough, because we are five people [in the household]. He [husband] delivers mail, he does not have a formal employment, when he is working, he is paid by the day (37 yrs., housewife).*

*Last year my husband lost his job, then we started to reduce [food expenditures] because we have to pay the electricity, water…(40 yrs., housewife).*

The availability of money in the households fluctuates depending on work-related payment schedules resulting in food access being reduced during several days or weeks of the month.

*The last days of the month, we do not have enough money to buy food, until the next month when we get paid (25 yrs., 3 children, housewife).*

*I have to cook less food to be able to reach [the end of the month], we do not always have food the last weeks of the month (21 yrs., 3 children housewife).*

#### Public services and household sanitation

The lack of urban infrastructure and exposure to environmental contaminants, constitute additional risks and stressors that increase the vulnerability of food insecure households. Some of the families lived in marginal enclaves within the urban boundaries, where public services like safe drinking water and electricity were not provided. The majority of the population in one settlement were immigrants from Bolivia who earned their livelihood by making bricks. In the area surrounding their homes, they could find clay and firewood, and they were able to construct ovens to fire-cook their production. Among the deficiencies in urban infrastructure in this settlement, the lack of safe drinking water was preeminent. When asked where the water they drink came from and whether it was clean, one participant responded:

*There is a wooden post standing there, there is a water overflow, from the water company. Sometimes the water [makes us sick] because it has insects, many things, it is dirty…but we strain it with a strainer or we tie a cloth to the pipe (21 yrs., housewife).*

A different group of families in this study lived in an area that used to be a dynamic industrial center developed around a government owned steel smelter. This productive activity was closed or down-sized through the privatization policy schemes of the 1990s and the area continues to be submerged in economic depression. Dwellers are exposed to the contaminants produced by longstanding industrial waste sites, to toxic fumes from factories and to the problems caused by open-air garbage dumping sites.

*That is a garbage dump, when the weather is hot and it rains, the smell… we have to lock ourselves up (47 yrs., 6 children, housewife).*

*There is also the smell from the paper mill and other factories, it depends on the direction of the wind, we wake up in the morning and the house is full of smoke (47 yrs., 6 children, housewife).*

#### Access to healthy food

The term ‘food deserts’ describes populated urban areas where residents do not have access to an affordable and healthy diet [47]. Food deserts may damage public health by restricting the availability and affordability of healthy foods [48, 49]. We asked the participants about their perceptions regarding the adequacy of food outlets in their neighborhood. Some reported that there were no food retail stores next to their home.

*There are no food stores here, there is one in [a nearby neighborhood], it is far. (21 yrs., 3 children, housewife).*

However, residents of another neighborhood in this study, reported that they shopped for vegetables and fruits in a nearby produce market. Open air markets are part of the informal economic system in Jujuy, and often offer lower prices and better fresh produce quality, than formal retail stores or supermarkets.

*No, the quality [of food] is not good here in the neighborhood. It is better to buy at the [open air] market because you can choose the produce, here they give you the vegetables in bad condition and at high price. (37 yrs., 5 children, housewife).*

*We buy in the [open air] market. They bring [the vegetables] directly from the farms, they bring fresh vegetables such as carrots, tomatoes, lettuce, potatoes, peas, all types of vegetables, also some fruit, apples, oranges, bananas, peaches and also pasta, corn meal, rice, oil, salt whatever is needed for cooking (31 yrs., one child, employed).*

The preceding sections described relevant aspects of the environmental context where the families in this study live. The following sections describe strategies used to cope with food insecurity.

### Negative coping strategies

Several strategies to cope with food insecurity have been consistently reported, like borrowing money to buy food, receiving assistance from family or neighbors, skipping meals or reducing food quantity and/or quality for some or all household members [13, 50]. Among strategies that can have a negative impact on the household nutritional quality we ascertained the practice of reducing food quantity and using cheap and less nutritious ingredients when running out of money.

*Yes, I have to reduce the ingredients to be able to cook…like buying less quantity. (40 years, 6 children, housewife).*

*We run out of things so we start eating less. (18 yrs., 1 child, student).*

*We buy cheaper things. Sometimes the vegetables are very expensive then we cannot use vegetables…we replace them with other things...when we do not have meat we eat hotdogs, or we make pasta with sauce (25 yrs., 2 children, housewife).*

*When we run out of money we cook whatever we have…pasta or rice stew (31 yrs., 1 child, employed).*

Among families with food insecurity some members, commonly adult women, reduce their food intake or skip meals to favor the more vulnerable, such as young children, or those who need strength to perform their job, like the adult men.

*My mother in law eats at work more than here [the home]…she saves the food for her children. She does not eat. (18 yrs., 1 child, student).*

*I eat less, so the children and also my husband can eat more, three or four days per week. (21 years, 3 children, housewife).*

### Buffering-protective resources

#### Extended family

Sixty-four percent of families in this study were multigenerational with newly constituted families incorporated to the parent’s home. The extended family has been described as a kinship group reaching beyond the nuclear family, with members sharing close mutual bonds and responsibilities and finding support and cooperation when needed [51]. We obtained participant’s accounts that underscore the functionality of extended familes for coping with food insecurity. Some of the strategies used can be instrumental in sustaining nutritional quality, like the contribution of income from members of the extended family.

*My brother in law has a job, so sometimes we have an extra income, on a weekend, so we have money for food…he was unemployed and not long ago he found a job. (33 yrs., 2 children, housewife).*

*My husband resorts to his father, his father lends him money (26 yrs., 1 child, employed).*

Another positive strategy consisted in buying food produce collectively among family members or neighbors.

*Sometimes families come from Bolivia with lots of children, what we do is, on Sundays we go [to the market] and buy bags of potatoes, pasta, eggs... we bake bread. (26 yrs., 2 children, employed)*.

#### The role of grandmothers

In the narratives of participants, the grandmothers emerged as central pillars in the household’s coping schemes. They contributed to the family income with their salaries or pensions and they took on leading roles in the administration of resources, in the organization of the household chores, in the provision of care for family members and in decision-making for food purchase and preparation.

*I live here with my baby, my father, my mother and my brothers. (31 yrs. 1 child, employed).*

*[in this home] there are one, two, three, four more families and my mother and my father (39 yrs., five children, employed).*

*This is not my house, it is my mother’s house (47 yrs. 4 children, housewife).*

*This is the house of my mother in law (18 yrs., 1 child, student).*

The collected narratives illustrate how extended families contribute to survival strategies, and underscore the role of grandmothers and even great grandmothers, as providers of material support. Responses to questions of how families paid for food, how they felt when the money was insufficient and where the income came from included the following:

-
*With my grandmother’s salary (19 yrs., 1 child, student).*
-
*I felt quite bad, because I share [the house] with my family, I am not alone and truly, I am living in my parent’s house, that is why I feel bad, because sometimes I cannot make it, with what I have to spend with my child, and I do not contribute to the household (30 yrs., 1 child, employed).*
-
*My mother pays for the food, she is retired and receives pension for being the mother of 7 children (39 yrs., 5 children, employed).*
-
*My mother provides a little bit of money, then my income, I also receive [government] assistance for my daughter, also a little, very little we can gather [by working] (47 yrs., 5 children, employed).*

In this study respondent’s narratives described the involvement of grandmothers in decision making regarding the type of food consumed in the household, and in taking responsibility for purchasing and preparing the food.

*My mother decides about the menu, what she is going to cook, all that, she decides, sometimes stew, sometimes breaded cutlet with rice or salad, corn meal, homemade pasta mmmm! Gnoquis all that (30 yrs., one child, employed).*

*We mostly buy food in the market; my mother goes with my father and my brother and we make one purchase all at once. (30 yrs., 1 child, employed)*

*No, I do not cook for my children; my mother, she cooks for everybody (39 yrs., 5 children, housewife).*

*My mother always cooks what we want to eat, we buy and she prepares it (24 yrs., 1 child, employed).*

*My mother decides what we are going to eat because she knows best, she knows what we need to buy (25 yrs., 2 children, student).*

Only Few participants reported that they prepared their own meals.

*I cook, during the day we decide what we are going to eat and I cook (40 yrs., 6 children, housewife).*

*Me and her [mother in law], I cook more often (18 yrs. 1 child, student).*

Women in this study sought refuge in their mother’s home even after moving out, and grandmothers who did not live with their daughters or daughters-in-law, continued to assist with obtaining and preparing foods and caring for their grandchildren. Co-habitation is not a necessary condition for the functionality of extended family support.

*I usually spend the night in my house over there, in the settlement, that is my house, but I do not have electricity, so I come here [her mother’s house] with my baby to wash clothes or watch TV because he gets bored….at night when his father arrives we go uphill.*

*When my mother is alone, I eat meals with her, otherwise I come here, to the house of my mother in law. (18 yrs., 1 child, student).*

## Discussion

The Eco-bio-developmental (EBD) framework and the vulnerability-assets approach, provide a basis for conceptualizing the significance of our results [52]. Beginning prenatally and extending into childhood and beyond, development is driven by an ongoing, inextricable interaction between biology (e.g., genetic predispositions) and ecology (e.g., the social and physical environment). Toxic stress in children, can result from strong, frequent, or prolonged activation of the body’s stress response systems in the absence of the buffering protection of a supportive adult relationship. Significant reductions in chronic disease could be achieved across the life course by decreasing the number and severity of adverse experiences that threaten the wellbeing of young children and by strengthening the protective relationships that help mitigate the harmful effects of toxic stress.

Food insecurity studies Latin America largely focus on examining frequency, risk factors and outcome data [21, 23, 24, 26, 28, 31, 50, 53–57], with few qualitative studies examining subjective experiences [15, 20, 21]. In this study, low income mothers of small children shared passages of their daily life shaped by the need to manage scarce resources. The type of adversities faced by these families included lack of urban infrastructure, water insecurity, environmental contaminants, and limited access to healthy food. These themes suggest the need to understand pathways in the link between compounded stressors, vulnerability and ill health, as well as the mitigating role of protective relationships. The results of this study highlight the importance of developing a more profound understanding of the compounding effect of environmental contaminants, the lack of household infrastructure and sanitation and inadequate food access and intake.

A growing concern in the public health field is the unequal exposure to environmental risk factors of disadvantaged population groups [58]. Environmental justice advocates that no population should bear disproportionate exposure to harmful environmental conditions [59, 60]. Research in this topic showed that low-income communities and people of non-hegemonic ethnic groups often suffer adverse and disproportionate exposure to environmental and occupational toxins. Inequities have been documented regarding exposure to lead, poor air quality, proximity to polluting industries, mining waste sites and nuclear plants, proximity to municipal waste sites, and contamination with pesticides [59, 61, 62]. It has also been ascertained that the population exposed to environmental contaminants may be more susceptible to health damage because genetic characteristics, socioeconomic factors and/or nutritional status, may increase the effect of environmental risks. Furthermore, children are especially vulnerable due to their early developmental stage.

Another stressor in our study was water insecurity, defined as having restrictions in the access to clean water that is estimated to affect more than 600 million people in the world [63]. A study showed that in a community of Ayacucho, Perú, 39% of households had food insecurity and none had access to safe drinking water [28]. Kirkpatrick and Tarasov [64] reported that low income families give up basic household services to purchase food. Another study conducted in Ethiopia, showed that water insecurity was associated to psychological stress [65]. Von Braun explored the relationship between health and sanitation and food safety, stating that urban food insecurity is a manifestation of a larger symptom –urban poverty—with multiple causes in which low incomes, food system deficiencies, sociocultural practices, and the health environment are linked [66].

Inequities in neighborhood food availability may limit the capacity to cope with insufficient household resources [60]. Research on the built environment seeks to understand the relationship between food retail access and dietary intake. Food price and accessibility can mediate the relationship between the built environment and nutrition [48, 67]. Early studies suggest that healthy foods may be more expensive and less available in poorer areas. Other studies show instead that healthy foods tend to be as, if not more, available in poorer areas and are lower in price [68]. Our results showed that food access may vary across low income neighborhoods, with some marginal areas lacking food stores and others benefiting from the presence of farmers markets that provide fresh fruits and vegetables. Monitoring healthy food availability in poor neighborhoods whether it is increased or limited, can contribute to improving nutritional programs.

Extended families constitute a primordial asset [69, 70] and strategies to optimize the material and social support provided by a kinship network may be integrated into food security programs [36]. The instrumental role of grandmothers in food insecure households is a salient aspect of the knowledge basis provided by this study. The benefits for the more vulnerable members of the household were apparent. The demands imposed on grandmothers by having to fulfill multiple provider roles, has not been a focus of inquiry in Latin America. Studies conducted in the northern hemisphere, showed that older adults living in food insufficient households had lower nutrient intakes and increased functional impairments, compared with other family members [71, 72]. Caregiving grandmothers tended to evaluate their health status as poor, to present dual pathologies, to delay health seeking and to undervalue their health problems. However, other studies found that although there was an initial decline in perceived health status, improvements occurred over time, and when grandchildren left the household, the grandmothers developed more functional limitations [73]. Greater life satisfaction has also been ascertained [74, 75]. The effect of caregiving in the life quality of grandmothers may depend on the form and level of caregiving, on individual characteristics, and on normative and cultural contexts [76].

Johnson et al., (2010) reported that the matrilineal influence (mothers, grandmothers, aunts) has a persistent influence on a family’s food choice. Ivers and Cullen [77] examined food insecurity with special consideration for women, recognizing their contribution to food production and preparation, and their role in society as child bearers and caregivers. Our study underscores the need to implement support and safety nets for caregiving grandmothers. Extended families maintain their relevance as social institutions in Latin American countries, and within this institution, grandmothers play a salient role. Nevertheless, we found no studies on the impact of the physical and emotional burden of caregiving, among grandmothers in this world region.

## Conclusions

This study generated insights on the experience of food insecurity among mothers with small children in Northwest Argentina. There were overarching economic system constrains that limited the capacity of households to attain a good quality of life, constituting determinants of ill health. These constrains were exemplified by the accounts of respondents: lack of employment opportunities, low paid and informal jobs, deficient housing infrastructure and environmental contamination. Thus, addressing food insecurity as a social issue is contingent upon resolving poverty as an underlying cause. The learnings provided by this study contribute to strategies that could, in the short term, attain changes at the community and household level, and contribute to ameliorate the health consequences of food insecurity, namely, developing collective advocacy venues for environmental and structural problems resolution, and developing safety nets and support for family pillars like grandmothers. Involvement of the primary health care level in low income communities could promote venues to reinforce buffering-protective resources and foster participatory reflection on how to address negative coping strategies.

## References

[1] Calvo E, Aguirre P. Crisis de la seguridad alimentaria en la Argentina y estado nutricional en una población vulnerable. Archivos Argentinos de Pediatria. 2005:77–90. http://www.scielo.org.ar/scielo.php?script=sci_arttext&pid=S0325-00752005000100015. Accessed 17 Mar 2017

[2] WHO Regional Office for Europe Programme, Security for N and F, ETC TN, Health WC for U. Urban and peri-urban food and nutrition action plan. Copenhagen; 2001. http://www.euro.who.int/__data/assets/pdf_file/0016/101626/E72949.pdf. Accessed 17 Mar 2017.

[3] Shonkoff JP, Garner AS, Siegel BS, Dobbins MI, Earls MF, Garner AS (2012). The lifelong effects of early childhood adversity and toxic stress. Pediatrics.

[4] Hales CN, Barker DJP (2001). The thrifty phenotype hypothesis. Br Med Bull Br Counc.

[5] Ke J, Ford-Jones E. Food insecurity and hunger: a review of the effects on children’s health and behaviour. Paediatr Child Heal. 2015;20 http://search.proquest.com/openview/a9f5317e00b248378b14587a9fdc43f6/1?pq-origsite=gscholar&cbl=2032237. Accessed 17 Mar 2017 10.1093/pch/20.2.89 PMC4373582 25838782

[6] Seligman HK, Bindman AB, Vittinghoff E, Kanaya AM, Kushel MB (2007). Food insecurity is associated with diabetes mellitus: results from the National Health Examination and nutrition examination survey (NHANES) 1999–2002. J Gen Intern Med.

[7] Seligman HK, Laraia BA, Kushel MB (2010). Food insecurity is associated with chronic disease among low-income NHANES participants. J Nutr.

[8] Tayie FA, Zizza CA (2009). Food insecurity and dyslipidemia among adults in the United States. Prev Med (Baltim).

[9] Food And Agriculture Organization. State of food insecurity in the world 2015: Food & Agriculture Org; 2015. http://www.fao.org/publications/card/es/c/c2cda20d-ebeb-4467-8a94-038087fe0f6e/. Accessed 17 Mar 2017

[10] Food and Agriculture Organization of the United Nations (FAO). Panorama of food and nutritional security in Latin America and the Caribbean 2014. Santiago de Chile; 2015. http://www.fao.org/3/a-i4018e.pdf. Accessed 18 Mar 2017.

[11] Franklin B, Jones A, Love D, Puckett S, Macklin J, White-Means S (2012). Exploring mediators of food insecurity and obesity: a review of recent literature. J Community Health.

[12] Birhane T, Shiferaw S, Hagos S, Mohindra KS (2014). Urban food insecurity in the context of high food prices: a community based cross sectional study in Addis Ababa, Ethiopia. BMC Public Health.

[13] Shariff ZM, Khor GL, Khor G, Tee E, Habicht J, Panigassi G (2008). Household food insecurity and coping strategies in a poor rural community in Malaysia. Nutr Res Pract.

[14] Kimani-Murage EW, Schofield L, Wekesah F, Mohamed S, Mberu B, Ettarh R (2014). Vulnerability to food insecurity in urban slums: experiences from Nairobi, Kenya. J Urban Heal.

[15] Bernal J, Frongillo EA, Herrera H, Rivera J (2012). Children live, feel, and respond to experiences of food insecurity that compromise their development and weight status in Peri-Urban Venezuela. J Nutr.

[16] Bhawra J, Cooke MJ, Hanning R, Wilk P, Gonneville SLH (2015). Community perspectives on food insecurity and obesity: focus groups with caregivers of Métis and off-reserve first nations children. Int J Equity Health.

[17] Connell CL, Lofton KL, Yadrick K, Rehner TA (2005). Children’s experiences of food insecurity can assist in understanding its effect on their well-being. J Nutr.

[18] Fenton C, Hatfield J, McIntyre L. A qualitative pilot studi of food insecurity among Maasai women in Tanzania. Pan Afr Med J. 13 http://www.ajol.info/index.php/pamj/article/view/85852. Accessed 17 Mar 2017 PMC3473967 23077702

[19] Vahabi M, Damba C (2013). Perceived barriers in accessing food among recent Latin American immigrants in Toronto. Int J Equity Health.

[20] Dean WR, Sharkey JR, Johnson CM, John J (2012). Cultural repertoires and food-related household technology within Colonia households under conditions of material hardship. Int J Equity Health.

[21] Lindsay AC, Ferarro M, Franchello A, de La Barrera R, Machado MMT, Pfeiffer ME (2012). Child feeding practices and household food insecurity among low-income mothers in Buenos Aires, Argentina. Cien Saude Colet.

[22] Coleman-Jensen A, Rabbitt MP, Gregory CA, Singh A (2016). Household food security in the United States in 2015.

[23] Weigel MM, Armijos RX, Racines M, Cevallos W (2016). Food insecurity is associated with undernutrition but not Overnutrition in Ecuadorian women from low-income urban neighborhoods. J Environ Public Health.

[24] de Haro-Mota R, Marceleño-Flores S, Bojórquez-Serrano JI, Nájera-González O, de Haro-Mota R, Marceleño-Flores S (2016). La inseguridad alimentaria en el estado de Nayarit, México, y su asociación con factores socioeconómicos. Salud Publica Mex.

[25] Schmeer KK, Piperata BA, Rodríguez AH, Torres VMS, Cárdenas FJC (2015). Maternal resources and household food security: evidence from Nicaragua. Public Health Nutr.

[26] Alberto Rivera Márquez Departamento de Atención la Salud J. Inseguridad alimentaria en el hogar y estado de nutrición en personas adultas mayores de México. Salud Publica Mex. 2014;56 suppl 1:S71–S78. http://www.scielosp.org/pdf/spm/v56s1/v56s1a11.pdf. Accessed 17 Mar 2017. 25649456

[27] Poblacion AP, Marín-León L, Segall-Corrêa AM, Silveira JA, Taddei JA de AC. (2014). Insegurança alimentar em domicílios brasileiros com crianças menores de cinco anos. Cad Saude Publica.

[28] Pillaca S, Villanueva M (2015). Evaluacion de la seguridad alimentaria y nutricional en familias del distrito de los Morochucos en Ayacucho, Perú. Rev Peru Med Exp Salud Publica.

[29] Fiszbein A, Giovagnoli PI, Giovagnoli PI (2004). Hambre en la argentina. Desarrollo Econ.

[30] Bolzán A, Mercer R (2009). Seguridad alimentaria y retardo crónico del crecimiento en niños pobres del norte argentino. Arch Argent Pediatr.

[31] Rosso MA, Wicky MI, Nessier MC, Meyer R (2015). Inseguridad alimentaria en la ciudad de Santa Fe: percepción de los ciudadanos. Salud Colect.

[32] Salvia A, Tuñón I, Musante B (2012). La Inseguridad Alimentaria en la Argentina.

[33] Shonkoff JP, Garner AS, Committee on psychosocial aspects of child and family health COECAADCASODABP, committee on early childhood, adoption, and dependent care, section on developmental and behavioral pediatrics (2012). The lifelong effects of early childhood adversity and toxic stress. Pediatrics.

[34] Garner A. Applying and ecobiodevelopmental framework to food insecurity: more thansimply food for thought. J Appl Res Child Informing Policy Child Risk. 2012;3 http://digitalcommons.library.tmc.edu/childrenatrisk/vol3/iss1/12. Accessed 25 Jun 2018

[35] Chilton M, Rabinovich J. The journal of applied research on children. J Appl Res Child Informing Policy Child Risk. 2012;3 http://digitalcommons.library.tmc.edu/childrenatrisk/vol3/iss1/3. Accessed 25 Jun 2018

[36] Moser CON (1998). The asset vulnerability framework: reassessing urban poverty reduction strategies. World Dev.

[37] Evans A (1991). Gender issues in rural household economics. IDS Bull.

[38] Attanasio OP, Székely M, Inter-American Development Bank. Portrait of the poor : an assets-based approach. Distributed by the Johns Hopkins University Press for the Inter-American Development Bank; 2001. https://books.google.com.ar/books?hl=es&lr=&id=avbXRh2AW4MC&oi=fnd&pg=PA45&dq=Poverty+and+assets+in+Bolivia:+What+role+does+social+capital+play&ots=sd-ChoziqF&sig=nAAFNLAuBd7Kyiiy9UQeOR5kd9I#v=onepage&q=Poverty and assets in Bolivia%3A What ro. Accessed 5 Jul 2018.

[39] Narain U, Gupta S, Van’t Veld K (2008). Poverty and the environment: exploring the relationship between household incomes, private assets, and natural assets. Land Econ.

[40] Martínez R, Palma A, Atalha E, Pinheiro AC (2009). Panorama social de America Latina. 2008.

[41] Romaguera D, Samman N, Farfán N, Lobo M, Pons A, Tur J. Nutritional status of the Andean population of Puna and Quebrada of Humahuaca, Jujuy, Argentina. Public Health Nutr. 2008;11 10.1017/S1368980007001061. 10.1017/S1368980007001061 17894917

[42] Alderete E, Bejarano I, Rodríguez A (2016). Beverage intake and obesity in early childhood: evidence form primary health care clients in Northwest Argentina. J Dev Orig Health Dis.

[43] Glaser BG, Strauss AL (1967). The discovery of grounded theory : strategies for qualitative research.

[44] Bickel G, Nord M, Price C, Hamilton W, Cook J (2000). Measuring food security in the United States guide to measuring household food security revised.

[45] Riessman CK (2008). Narrative methods for the human sciences.

[46] Barbour RS (2001). Education and debate checklists for improving rigour in qualitative research: a case of the tail wagging the dog? Checklists in quantitative research. BMJ.

[47] Cummins S, Macintyre S (2002). “Food deserts”—evidence and assumption in health policy making. BMJ.

[48] Black JL, Macinko J (2008). Neighborhoods and obesity. Nutr Rev.

[49] Sallis JF, Glanz K (2009). Physical activity and food environments: solutions to the obesity epidemic. Milbank Q.

[50] Piperata BA, Schmeer KK, Hadley C, Ritchie-Ewing G (2013). Dietary inequalities of mother–child pairs in the rural Amazon: evidence of maternal-child buffering?. Soc Sci Med.

[51] Madsen W (1964). Mexican-Americans of South Texas.

[52] Shonkoff J, Garner A, Siegel B, Dobbins M. The lifelong effects of early childhood adversity and toxic stress. Pediatrics. 2012; http://pediatrics.aappublications.org/content/129/1/e232.short. Accessed 17 Mar 2017 10.1542/peds.2011-2663 22201156

[53] Lindemann IL, Oliveira RR, Mendoza-Sassi RA (2016). Dificuldades para alimentação saudável entre usuários da atenção básica em saúde e fatores associados. Cien Saude Colet.

[54] Sperandio N, Priore SE, Sperandio N, Priore SE (2015). Prevalence of household food insecurity and associated factors among Bolsa Família program families with preschool children in Viçosa, Minas Gerais State, Brazil. Epidemiol e Serviços Saúde.

[55] Bonfin de Sabóia RC, Melo dos Santos M. Prevalence of food insecurity and associated factors in households covered by the Family Health Strategy in Teresina-PI, Brazil. 2012;24 10.5123/S1679-49742015000400017.

[56] Bortolini GA, Vitolo MR, Gubert MB, Santos LMP (2015). Iniquidades sociais influenciam a qualidade e a diversidade da dieta de crianças brasileiras de 6 a 36 meses. Cad Saude Publica..

[57] Cavalcante Martins M, dos Santos AJ, Bezerra Dantas K, Moraes de Sabino LM, Santos Alves MD, Barbosa Ximenes L (2015). Food consumption among families of pre-school children in situation of food (in)security. Cienc y enfermería.

[58] Brulle RJ, Pellow DN (2006). Environmental justice: human health and environmental inequalities. Annu Rev Public Health.

[59] Lee C. Environmental justice: building a unified vision of health and the environment. Environ Health Perspect. 2002;110 https://www.ncbi.nlm.nih.gov/pmc/articles/PMC1241156/pdf/ehp110s-000141.pdf. Accessed 17 Mar 2017 10.1289/ehp.02110s2141 PMC1241156 11929721

[60] Hilmers A, Hilmers DC, Dave J (2012). Neighborhood disparities in access to healthy foods and their effects on environmental justice. Am J Public Health.

[61] Mohai P, Saha R (2006). Reassesing racial and socioeconomic disparities in environmental justice research. Demography.

[62] Loopstra R, Tarasuk V (2013). Severity of household food insecurity is sensitive to change in household income and employment status among low-income families. J Nutr.

[63] WHO/UNICEF Joint Water Supply and Sanitation Monitoring Programme., World Health Organization, UNICEF. Progress on sanitation and drinking water. 2015 update and MDG assessment. https://books.google.cl/books?hl=es&lr=&id=KFA0DgAAQBAJ&oi=fnd&pg=PA1&dq=Progress+on+drinking+water+and+sanitation+2015+update&ots=l5Z5eJY4n5&sig=p2xxGHeEtUfkFz_F-YFQ0h2n_wI#v=onepage&q=Progress on drinking water and sanitation%2C 2015 update&f=false. Accessed 17 Jul 2018.

[64] Kirkpatrick SI, Tarasuk V (2008). Food insecurity is associated with nutrient inadequacies among Canadian adults and adolescents. J Nutr.

[65] Stevenson EGJ, Ambelu A, Caruso BA, Tesfaye Y, Freeman MC (2016). Community water improvement, household water insecurity, and Women’s psychological distress: an intervention and control study in Ethiopia. PLoS One.

[66] Braun J von. Urban food insecurity and malnutrition in developing countries : trends, policies, and research implications. IFPRI; 1993. https://books.google.com.ar/books?hl=es&lr=&id=52KddYfOpXsC&oi=fnd&pg=PR7&dq=von+braun+and+urban&ots=cXo8WnWvoP&sig=etbFgOepClolIia9mgUh23iPK0A#v=onepage&q=von braun and urban&f=false. Accessed 25 Jun 2018.

[67] Frank L, Kerr J, Saelens B, Sallis J, Glanz K, Chapman J (2008). Food outlet visits, physical activity and body weight: variations by gender and race-ethnicity. Br J Sports Med.

[68] White M (2007). Food access and obesity. Obes Rev.

[69] Keefe S (1984). Real and Ideal Extended Familism Among Mexican Americans and Anglo Americans: On the Meaning of “Family Ties.”. Hum Organ.

[70] Keefe S, Padilla A, Carlos M (1979). The Mexican-American Extended Family As An Emotional Support System. Hum Organ.

[71] Lee JS, Frongillo EA (2001). Nutritional and health consequences are associated with food insecurity among U.S. elderly persons. J Nutr.

[72] Rose D, Oliveira V (1997). Nutrient intakes of individuals from food-insufficient households in the United States. Am J Public Health.

[73] Hughes ME, Waite LJ, Lapierre TA, Luo Y (2007). All in the family: the impact of caring for grandchildren on grandparents’ health. J Gerontol B Psychol Sci Soc Sci.

[74] Davidhizar R, Bechtel GA, Woodring BC (2000). The changing role of grandparenthood. J Gerontol Nurs.

[75] Xu L, Wu B, Chi I, Hsiao H-Y (2012). Intensity of grandparent caregiving and life satisfaction among rural Chinese older adults. Fam Community Health.

[76] Chen F, Liu G (2012). The health implications of grandparents caring for grandchildren in China. J Gerontol Ser B Psychol Sci Soc Sci.

[77] Ivers LC, Cullen KA (2011). Food insecurity: special considerations for women. Am J Clin Nutr.

